# *Clostridioides difficile* infection cases and the causative strains in a large Chinese tertiary hospital in 2023–2024

**DOI:** 10.3389/fmicb.2026.1749145

**Published:** 2026-02-11

**Authors:** Xin Lu, Yiqi Liang, Yu Feng, Tingting Wang, Zhiyong Zong, Xiaohui Wang

**Affiliations:** 1 Center of Infectious Diseases, West China Hospital, Sichuan University, Chengdu, China; 2 Division of Infectious Diseases, State Key Laboratory of Biotherapy, Chengdu, China; 3 Center for Pathogen Research, West China Hospital, Sichuan University, Chengdu, China; 4 Department of Laboratory Medicine, Clinical Laboratory Medicine Research Center, West China Hospital, Sichuan University, Chengdu, China; 5 Sichuan Public Health General Clinical Center, Jincheng Hospital of West China Hospital, Sichuan University, Chengdu, China

**Keywords:** antimicrobial resistance, *Clostridioides difficile*, molecular epidemiology, multilocus sequence typing, whole-genome sequencing

## Abstract

**Background:**

*Clostridioides difficile* is a global urgent-threat pathogen, with prevalence and clinical impact varying over time and across regions. This study aims to elucidate the landscape of *C. difficile* infection (CDI) and its causative strains in Southwest China after the COVID-19 pandemic.

**Methods:**

This retrospective study enrolled CDI patients hospitalized between June 2023 and May 2024 at a large tertiary hospital in Southwest China, who were diagnosed via glutamate dehydrogenase (GDH) combined with toxin A/B testing. Toxigenic isolates from positive stool samples were submitted for antimicrobial susceptibility testing and whole-genome sequencing (WGS). Multilocus sequence typing (ST), identification of resistance determinants, and core-genome SNP (cgSNP) analysis were integrated with clinical data.

**Results:**

One hundred fifty-seven CDI patients were identified among 2,917 suspected patients with diarrhea. 67.5% of patients were male, 51.0% were ≥65 years, 20.4% had severe CDI, and two patients were fulminant. All 109 isolates remained susceptible to vancomycin and fidaxomicin. The moxifloxacin resistance rate reached as high as 56.0%, primarily driven by *gyrA* Thr82Ile and *gyrB* Asp426Val/Ser366Ala mutations. ST3 (23.9%) remains the most prevalent clone. ST81 (18.3%), all resistant to moxifloxacin and 20% resistant to metronidazole, has replaced ST37 (7.3%) as the second-most prevalent clone. ST5 (4.6%) was the main prevalent clone producing the binary toxin, and no ST1/RT027 was identified. The *cfr(B)* resistance gene was first detected in a ST54 isolate from China. CgSNP analysis identified 4 genetically highly related ST3 clone groups (≤2 SNPs within 124 days).

**Conclusion:**

In the post-pandemic era, the clinical burden of CDI in Southwest China cannot be overlooked. ST81 with high-level fluoroquinolone resistance has increased significantly and deserves more attention. Integration of data on clinical cases and their pathogenic strains through sustained clinical case monitoring, genomic surveillance of isolates, and antimicrobial resistance (AMR) pattern surveys provides early warning for future clonal dissemination and supports clinical management and public health decisions.

## Introduction

*Clostridioides difficile* is a Gram-positive spore-forming bacillus that causes pathogenicity by producing toxin A and toxin B. Some strains can also produce a binary toxin, CDT, which significantly enhances virulence (Di Bella et al., 2024). From a global public health perspective, the Centers for Disease Control and Prevention (2019) of the United States has classified it as an “urgent threat pathogen”. Globally, systematic reviews and disease burden studies indicate that the burden of CDI continues to rise in regions with high Socio-Demographic Index and among elderly populations, although significant spatiotemporal distribution variations exist across different areas (Xia et al., 2025).

Over the past two decades in North America and Europe, the hypervirulent strain ST1/RT027, which caused large-scale outbreaks in the mid-2000s, gradually declined, while RT014/020, RT002, and ST11/RT078 increased instead (2024) (European Centre for Disease Prevention and Control, 2024). Over the past decade in China, researchers and clinicians have increasingly focused on CDI, but most of the research has come from Beijing and eastern China, with limited data from other regions. A systematic review and meta-analysis of the literature, covering the sampling years between 2009 and 2020, reported an overall CDI detection rate of 11.4% (2,696/26,852) (Wen et al., 2023). However, only 4.1% of the data came from Southwest China, excluding Sichuan and Henan provinces, which are among the Top 5 provinces by population. To date, a nationwide surveillance network for *C. difficile*, similar to those in Europe or North America, has not been established.

The COVID-19 pandemic has imposed profound challenges on global health systems, including those related to CDI. During the pandemic, a bundle of robust infection prevention and control measures was implemented, influencing the epidemiology of CDI (Vendrik et al., 2022). Interestingly, the incidence of CDI rose in one study (Karampatakis et al., 2023) but fell in another (Ponce-Alonso et al., 2021). The extensive use of broad-spectrum antibiotics and the escalating issue of AMR severely disrupted gut microbiota and increased the risk of CDI (Markovska et al., 2023). Additionally, reports indicated a rise in severe CDI cases during the mid-to-late stages of the pandemic, potentially linked to delayed medical consultation and diagnosis (Vendrik et al., 2022). These dual pressures may have accelerated adaptive evolution in *C. difficile*. For instance, studies have identified the emergence of a new epidemic strain, RT176, in central Europe during the pandemic (Krutova et al., 2025). Therefore, the long-term impacts of the pandemic on the dynamics of evolution and AMR of *C. difficile* require further monitoring and research. In December 2022, in China, the “zero-COVID” policy was relaxed, and both population-wide screening and the digital travel code were canceled. Characteristic changes in CDI cases and *C. difficile* strains after the pandemic are unclear.

In this study, we analyzed clinical data from CDI patients admitted 6 months after policy relaxation to a 5,000-bed tertiary hospital that also serves as a national center for managing complex cases and critical patients transferred from all hospitals in Southwest China. *C. difficile* isolates were recovered from fecal samples of these patients and subjected to WGS. By combining clinical data and genomic analyses, this study seeks to clarify the profiles of *C. difficile* and CDI cases after the pandemic, offering healthcare professionals additional insights to enhance future monitoring and prevention of CDI.

## Method

### Study design and case enrollment

This retrospective study enrolled CDI cases from inpatients whose onset occurred between June 1, 2023, and May 31, 2024, at one of the largest tertiary hospitals in Southwest China, which serves over 5,000,000 outpatients and 250,000 inpatients annually. Clinical data were retrieved from the electronic medical record system. Two physicians specialized in infectious diseases independently reviewed the case and confirmed the diagnosis. According to the clinical practice guidelines recommended by the Infectious Diseases Society of America and the Society for Healthcare Epidemiology of America (Kelly et al., 2021), CDI was defined as cases with diarrhea (≥3 unformed stools within 24 h) without any identifiable cause, along with positive results for both GDH and toxins A/B using the C. DIFF QUIK CHEK COMPLETE^®^ assay (TechLab). Severe CDI cases were defined as the presence of a peripheral white blood cell (WBC) count ≥15 × 10^9^/L or a serum creatinine level ≥1.5 times the premorbid baseline. Severe CDI cases accompanied by hypotension, shock, ileus, or megacolon are clinically classified as fulminant infection. This study was approved by the Biomedical Ethics Committee of West China Hospital, Sichuan University, and informed consent was waived.

### Strain isolation and identification

If both the GDH and toxin A/B tests were positive, the leftover feces were submitted for anaerobic culture at 37 °C for 48 h, using cycloserine-cefoxitin fructose agar (CCFA, Oxoid, United Kingdom) supplemented with 500 mg/L D-cycloserine and 16 mg/L cefoxitin. Characteristical *C. difficile* colonies were passaged onto fresh blood agar plates supplemented with 5% defibrinated sheep blood and incubated anaerobically for purification. Isolates were identified using matrix-assisted laser desorption/ionization time-of-flight mass spectrometry (MALDI-TOF MS; Bruker Daltonics) and subsequently confirmed by PCR with validated primers for the housekeeping gene *tpi* and the toxin genes *tcdA*, *tcdB*, *cdtA*, and *cdtB* (Lemee et al., 2004; Persson et al., 2008).

### Antimicrobial susceptibility testing

According to the recommendations from the Clinical and Laboratory Standards Institute (CLSI) (Carpenter et al., 2018; Mathers et al., 2025) and the European Committee on Antimicrobial Susceptibility Testing (2025; Mathers et al., 2025), the minimum inhibitory concentrations (MICs) of fidaxomicin, metronidazole, moxifloxacin, and vancomycin were determined using the agar dilution method and interpreted based on the breakpoints. *C. difficile* ATCC 700057 and *Bacteroides fragilis* ATCC 25285 were used as quality controls.

### Whole-genome sequencing

Bacterial genomic DNA was extracted using the QIAamp DNA Mini Kit (Qiagen). Libraries were prepared with the NEBNext Ultra II DNA Library Prep Kit (New England Biolabs) and size-selected (350 ± 50 bp) using SPRIselect beads (Beckman Coulter). Paired-end sequencing (2 × 150 bp) was performed on the Illumina NovaSeq 6000 platform. Adapter-trimmed reads were subjected to quality filtering (Phred score ≥30, 4-bp sliding window) using Trimmomatic v0.39 (Bolger et al., 2014). Genome assembly was conducted using SPAdes v3.15.5 (Bankevich et al., 2012) in “isolate” mode with SeqKit v2.8.2 (Shen et al., 2016), and coverage was down-sampled to 150×. All genome sequences are deposited in NCBI under the BioProject accession PRJNA1364752; Supplementary Table S1 lists the accession numbers for each genome sequence.

### Molecular typing and phylogenetic analysis

Multilocus sequence type (MLST) was assigned according to various alleles of seven housekeeping genes (*adk*, *atpA*, *dxr*, *glyA*, *recA*, *sodA*, *tpi*) in the PubMLST database (Jolley et al., 2018). Core-genome single-nucleotide polymorphisms (cgSNPs) were determined using Snippy v4.6.0^1^ against the *C. difficile* 630 reference genome (GenBank: AM180355.1), with thresholds of mapping quality ≥30 and allele frequency ≥90%. Recombinant regions were masked using Gubbins v2.4.1 (Croucher et al., 2015). Pairwise SNP distances were calculated via snp-dists v0.8.2^2^. Suspected transmission clusters were defined as isolates differing by 0–2 cgSNPs within a 124-day interval (Eyre et al., 2013), and additional data of the associated patients were collected via the electronic medical record system. Minimum spanning trees (MSTs) were constructed from the pairwise cgSNP distance matrices using Python v3.11.2 and NetworkX v3.6.1 (minimum_spanning_tree) (Hagberg et al., 2008), and the resulting networks were imported into Cytoscape v3.10.4 for visualization (Shannon et al., 2003). Phylogenies were inferred using RAxML-NG v1.1.0 (GTR + G model, 1,000 bootstrap replicates) (Kozlov et al., 2019) and visualized with tvBOT v2.6.1 (Xie et al., 2023).

### Detection of resistance genes, virulence genes, and plasmids

The AMR genes and virulence factors were predicted using AMRFinderPlus (Feldgarden et al., 2021) and the Virulence Factor Database (VFDB), respectively, with thresholds of >90% for both coverage and identity. Mutations in *gyrA*/*gyrB* were identified by comparison with *C. difficile* 630 (GenBank: CP010905.2). For metronidazole resistance genes and plasmids, the *nimB* gene, its promoter sequence mutations and the presence of pCD-METRO were analyzed as outlined in prior studies (Boekhoud et al., 2020; Stojanovic et al., 2025). Regarding two studies (Mansfield et al., 2020; Shen et al., 2020) establishing TcdB subtyping schemes show consistency in the main TcdB subtypes (TcdB1–4) but differ in some minor subtypes (TcdB5–8), TcdB subtypes in this study were assigned based on amino-acid sequences translated from *tcdB*, using the DiffBase nomenclature (Mansfield et al., 2020).

### Statistical analysis

All statistical analyses were performed using SPSS Statistics version 28.0 (IBM Corp., Armonk, NY, United States). Continuous variables were assessed for normality with the Shapiro-Wilk test and presented as mean ± standard deviation (SD) or median with interquartile range (IQR), as appropriate.

## Results

### Clinical characteristics of CDI cases

**Figure 1:**
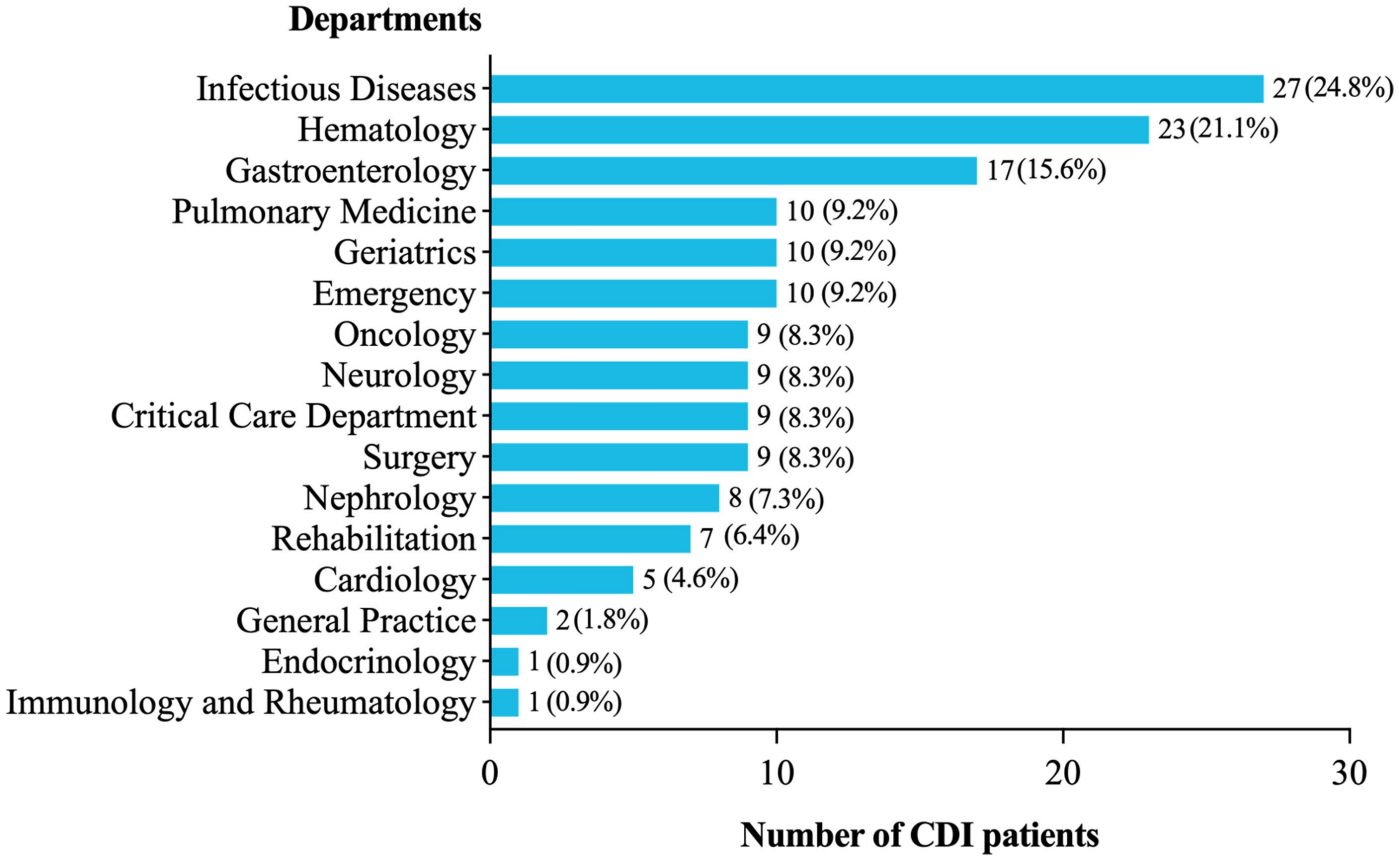
Distribution of CDI patients in different wards.

A total of 2,917 suspected patients with diarrhea underwent the GDH and toxin A/B test. Among them, 157 patients (5.4%, 157/2,917) were identified as CDI cases with the initial episode, and 109 non-duplicated toxigenic *C. difficile* strains were successfully isolated from their first submitted feces. As illustrated in Figure 1, many CDI cases were observed in the Department of Infectious Diseases (17.2%, 27/157), the Hematology Department (14.6%, 23/157), and the Gastroenterology Department (10.8%, 17/157), while only 5.7% in the ICU (9/157).

As shown in Table 1, patients ranged in age from 15 to 95 years, and 51.0% (80/157) were 65 years or older. Males accounted for 67.5% (106/157), and no pregnant patients were included. About comorbidities, many CDI patients had solid tumors (21.0%, 33/157), hematological malignancies (14.0%, 22/157), and diabetes (20.4%, 32/157). The top 3 risk medications used within 8 weeks prior to the onset of CDI were proton pump inhibitors (PPIs) (56.7%, 89/157), carbapenems (54.1%, 85/157), and β-lactam/β-lactamase inhibitor combinations (48.4%, 76/157). Notably, 20.4% (32/157) of CDI patients suffered severe infections, and even two patients with fulminant infection developed hypotensive shock. Regarding clinical outcomes, 7 patients (4.5%) died within 30 days of onset due to deteriorated comorbid conditions, and 25 patients (15.9%) gave up medical advice and voluntarily left the hospital.

**Table 1:** Clinical characteristics of 157 patients infected with toxigenic *C. difficile*.

Demographic information	Value (IQR/%)
Age	66 (50.5–76)
≥65 years	80 (51.0%)
75–89 years	39 (24.8%)
≥90 years	6 (3.8%)
Male	106 (67.5%)
BMI (kg/m^2^)	21.05 (18.93–24.53)
Comorbidities	
Solid tumor	33 (21.0%)
Diabetes	32 (20.4%)
Hematologic malignancy	22 (14.0%)
Chronic kidney disease	20 (12.7%)
History of gastrointestinal surgery	18 (11.5%)
Organ transplantation	12 (7.6%)
Inflammatory bowel disease	10 (6.4%)
Medication use	
Antimicrobial agents	
Carbapenems	85 (54.1%)
β-lactam and β-lactamase inhibitor combinations	76 (48.4%)
Quinolones	38 (24.2%)
Third/fourth generation cephalosporins	17 (10.8%)
Tetracyclines and glycylcyclines	16 (10.2%)
Trimethoprim-sulfamethoxazole	9 (5.7%)
Macrolides and lincosamides	3 (1.9%)
Other medications	
Proton pump inhibitors	89 (56.7%)
Severity classification	
Non-severe infection	125 (79.6%)
Severe infection	30 (19.1%)
Fulminant infection	2 (1.3%)
Post-CDI outcomes	
Recurrence after this episode	2 (1.3%)
Transfer to ICU	14 (8.9%)
Death within 30 days	7 (4.5%)
Discharge against medical advice	25 (15.9%)

BMI, Body Mass Index; CDI, *C. difficile* infection; ICU, intensive care unit.

### Genetic characteristics of toxigenic *C. difficile* isolates

**Figure 2:**
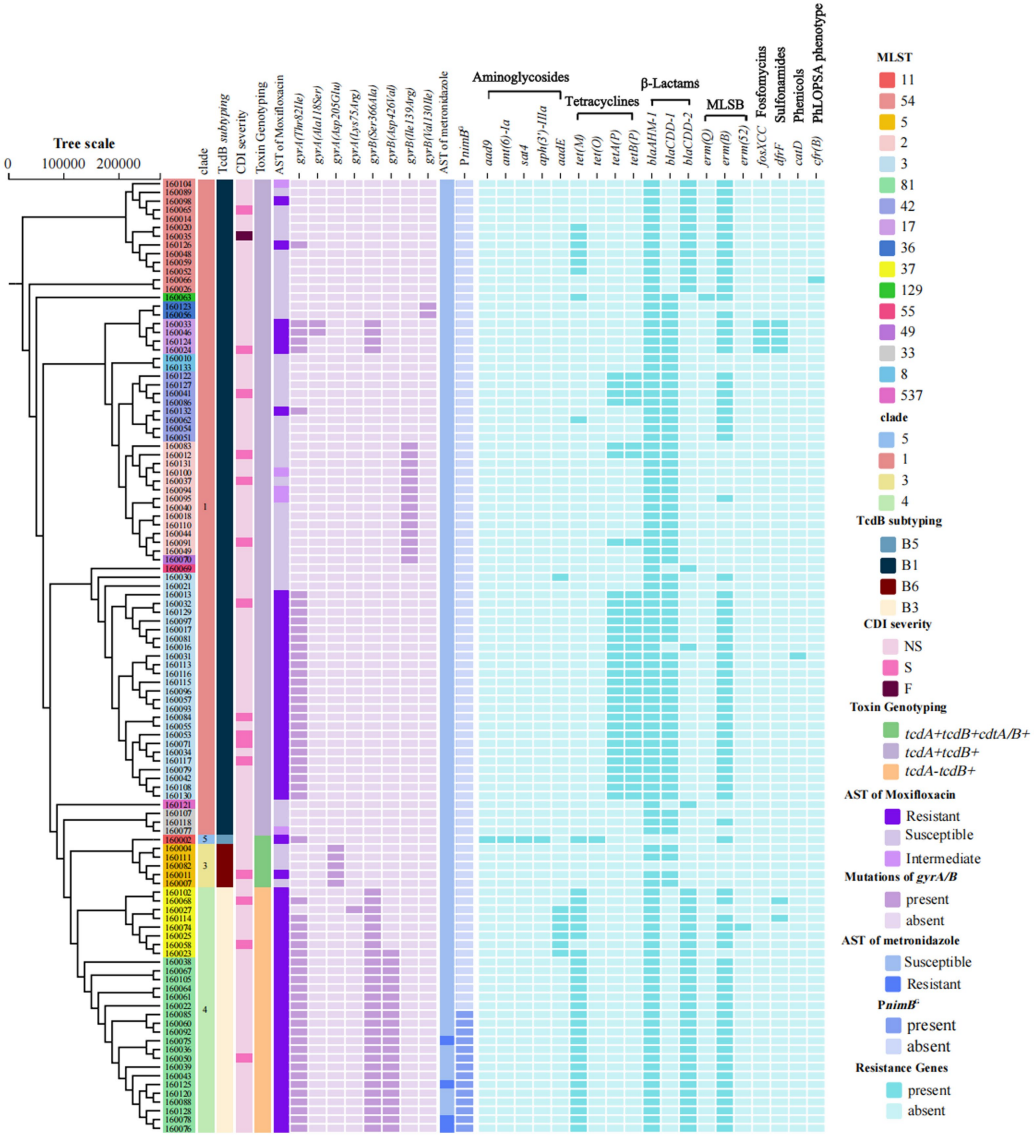
Phylogenetic tree of clinical toxigenic *C. difficile* isolates. From left to right, strain ID background colors denote MLST; followed by the distribution of clades, TcdB subtypes, CDI severity, toxin genotyping, susceptibility to moxifloxacin, amino acid substitutions in GyrA and GyrB, susceptibility to metronidazole, mutations in the *nimB* gene and its promoter sequence, and AMR genes. MLST, multilocus sequence typing; CDI, *C. difficile* infection; NS, non-severe infection; S, severe infection; F, fulminant infection; P*nimB*^G^, a T → G nucleotide mutation in the promoter of *nimB* gene; MLSB, Macrolide-lincosamide-streptogramin B; PhLOPSA, Phosphomycin, Lincosamides, Oxazolidinones, Phenicols, Streptogramins, Aminoglycosides.

**Figure 3:**
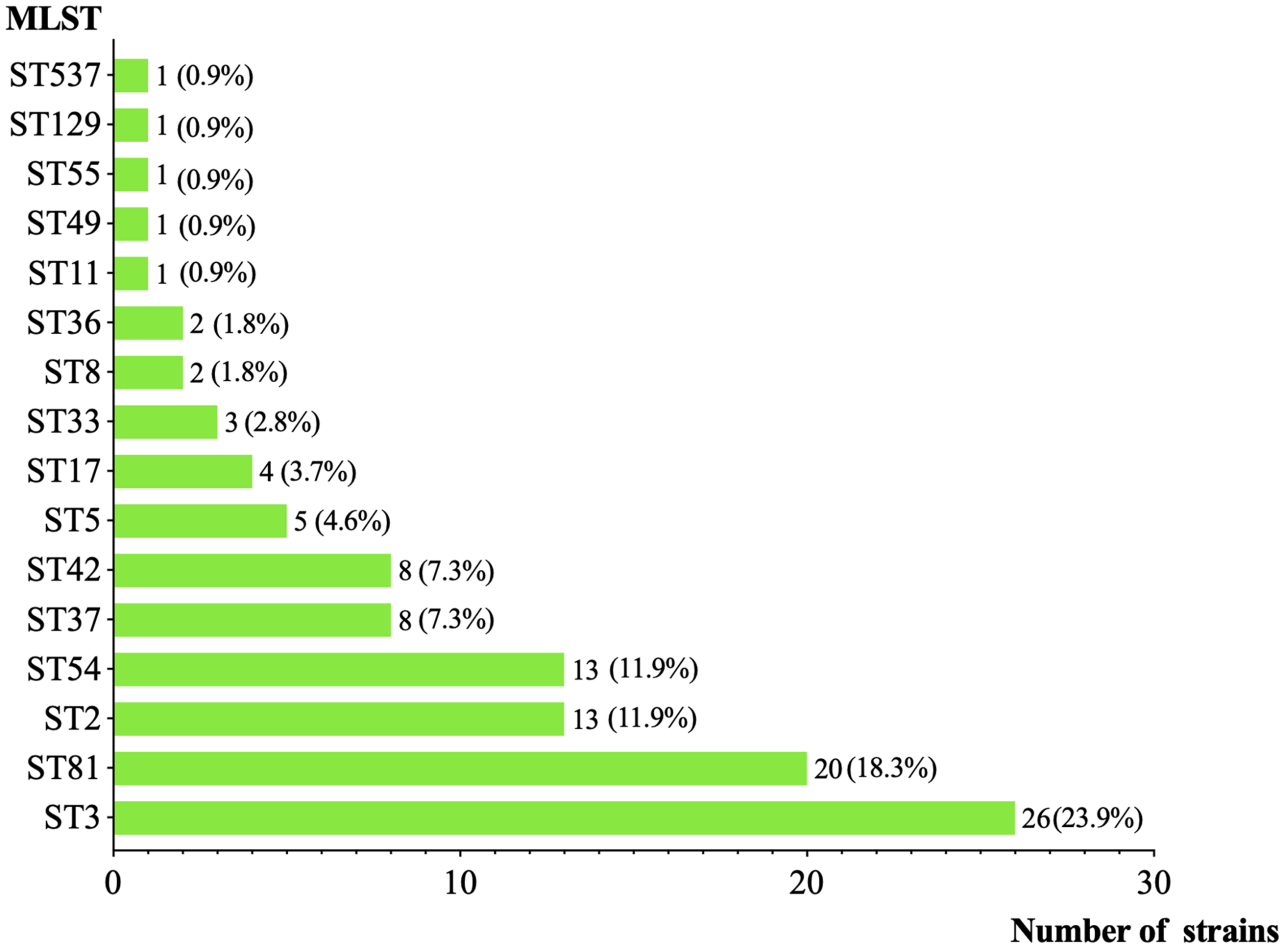
Distribution of MLST types.

As shown in Figure 2, among the 109 toxigenic *C. difficile* isolates, the most prevalent toxin genotype was *tcdA* + *tcdB* + *cdtA/B*− (68.8%, 75/109), primarily comprising strains of ST3, ST2, and ST54. This was followed by *tcdA* − *tcdB* + *cdtA/B*− (25.7%, 28/109), consisting of ST81 and ST37. Only 6 isolates (5.5%, 6/109) were *tcdA* + *tcdB* + *cdtA/B+*, specifically one ST11 and five ST5 strains. ST revealed ST3 as the most prevalent clone (23.9%, 26/109) (Figure 3), followed by ST81 (18.9%, 20/109), whereas ST37 accounted for only 7.3% (8/109). Notably, no isolate was detected that belonged to a hypervirulent ST1/RT027 clone. Phylogenetic clustering placed the isolates into clades 1, 3, 4, and 5, with clade 1 being most prevalent (68.8%, 75/109). Neither clade 2 nor cryptic lineages were identified. Further subtyping of TcdB identified four primary subtypes, which correlated consistently with clade distribution: clade 1 strains harbored TcdB1 (68.8%, 75/109), clade 4 carried TcdB3 (25.7%, 28/109), clade 5 exhibited TcdB5 (0.9%, 1/109), and clade 3 contained TcdB6 (4.6%, 5/109).

### Antimicrobial resistance of toxigenic *C. difficile* isolates

**Figure 4:**
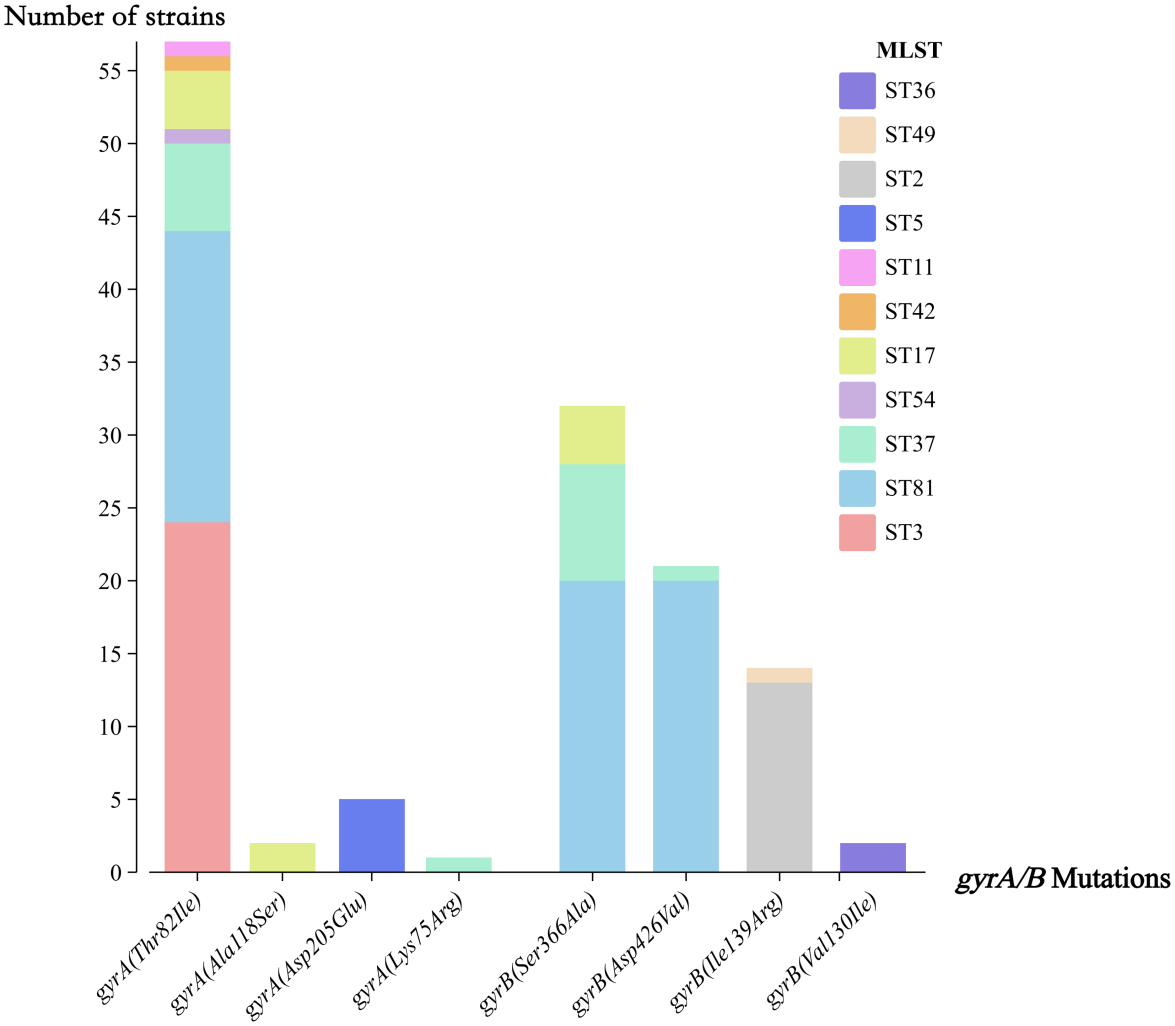
Distribution of *gyrA/B* mutations across strain types.

Agar dilution susceptibility testing revealed that all 109 toxigenic isolates were susceptible to vancomycin (MIC range, 0.25–2 mg/L) and fidaxomicin (MIC range, 0.015–0.5 mg/L), while 56.0% (61/109) exhibited resistance to moxifloxacin (Table 2). As shown in Figure 2, all ST37 and ST81 strains were resistant to moxifloxacin. Meanwhile, 52.3% (57/109) of the strains carried a Thr82Ile substitution in the *gyrA*, while 29.4% (32/109) had a Ser366Ala substitution in the *gyrB*. Among the mutations in *gyrA* Thr82Ile, ST3 (42.1%, 24/57) and ST81 (35.1%, 20/57) accounted for the highest proportions. For mutations in *gyrB*, ST81 represented the majority of cases with Ser366Ala (62.5%, 20/32) and Asp426Val (95.2%, 20/21), respectively (Figure 4). With respect to metronidazole resistance determinants, the plasmid pCD-METRO was not detected in any of the strains, but a T → G nucleotide mutation in the promoter (P*nimB*^G^) was identified in 70.0% (14/20) ST81 strains. Among them, 4 ST81 strains were resistant to metronidazole (MIC = 8 mg/L).

**Table 2:** Antimicrobial susceptibility of 109 toxigenic *C. difficile* isolates.

Antimicrobial agent	Breakpoint *R* (mg/L)	MIC range (mg/L)	MIC_50_ (mg/L)	MIC_90_ (mg/L)	Resistance rate
Fidaxomicin	>0.5	0.015–0.5	0.125	0.25	0 (0.0%)
Metronidazole	>2	0.125–8	0.5	2	4 (3.7%)
Moxifloxacin	≥8	1–256	16	256	61 (56.0%)
Vancomycin	>2	0.25–2	0.5	1	0 (0.0%)

Using AMRFinderPlus, 19 AMR genes (ARGs) were identified, conferring resistance to aminoglycosides, tetracyclines, β-lactams, macrolides, lincosamides, streptogramins, chloramphenicol, trimethoprim, and fosfomycin (Figure 2). Among these isolates, 74.3% (81/109) carried the *erm(B)* gene and one isolate each harbored *erm(Q)* and *erm(52)*, which mediate resistance to macrolide-lincosamide-streptogramin B (MLSB) antibiotics. The *bla*_AHM-1_ gene was present in nearly all strains (98.2%, 107/109). Additionally, *bla*_CDD-1_ and *bla*_CDD-2_ were detected in 57.8% (63/109) and 40.4% (44/109) of the strains, respectively, both of which are associated with β-lactam resistance. Tetracycline resistance-related genes were also frequently detected. 28.4% (31/109) of the strains carried both *tetA(P)* and *tetB(P)*, 33.0% (36/109) contained *tet(M)*, and one strain was positive for *tet(O)*. Aminoglycoside resistance-related genes were primarily distributed in specific sequence types: one ST11 strain simultaneously carried *aad9*, *ant(6)-Ia*, *sat4*, and *aph(3′)-IIIa* without *aadE*. Conversely, *aadE* was detected in 75.0% (6/8) ST37 strains and one ST3 strain. Notably, the *cfr(B)* gene, related to linezolid resistance, was identified in one ST54 strain.

### Clone relatedness and clonal dissemination assessment

**Figure 5:**
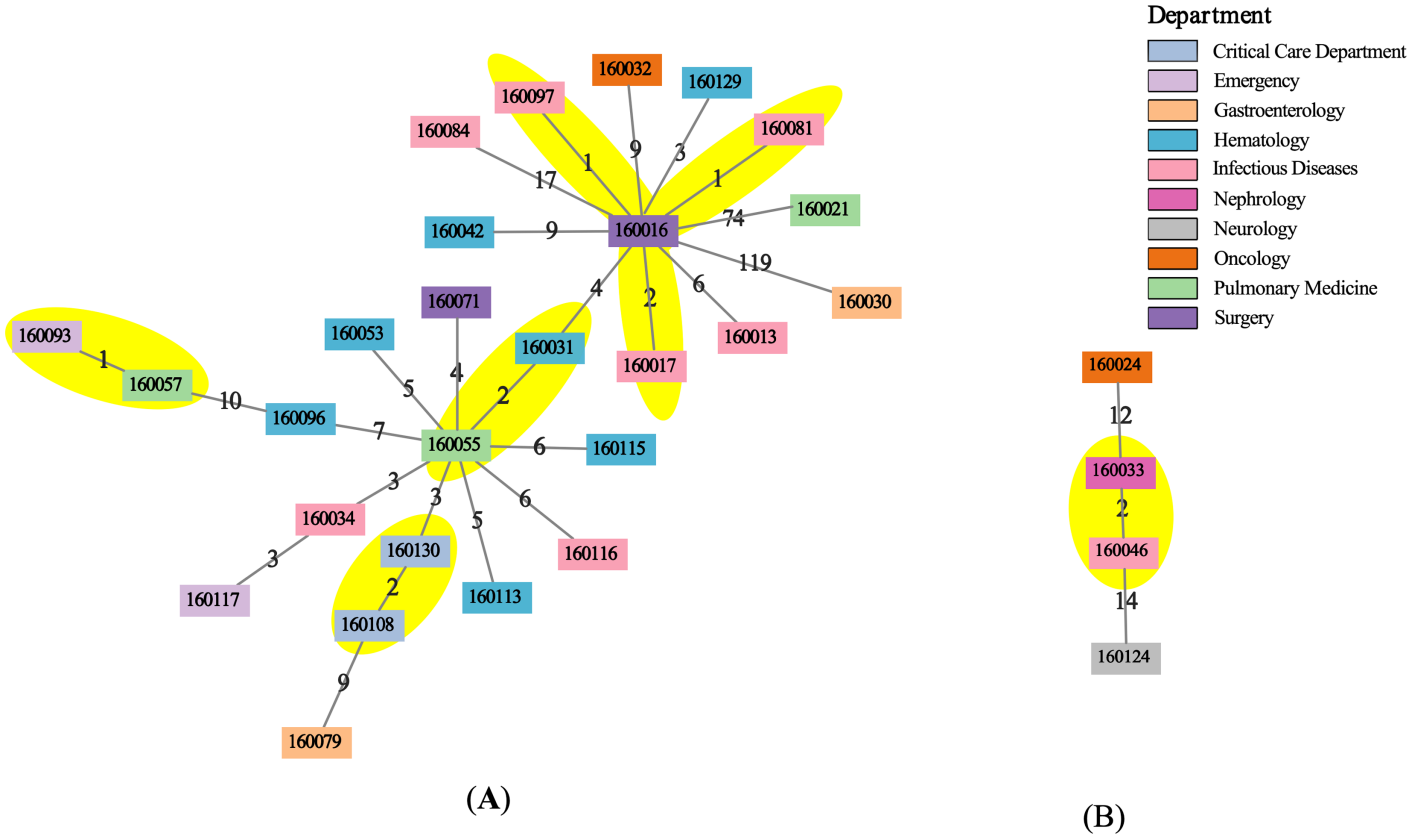
MSTs of ST3 and ST17 isolates. MSTs for **(A)** ST3 and **(B)** ST17 were generated from pairwise cgSNP distances. Nodes represent isolates and are colored by hospital department. Edge labels indicate integer SNP distances between connected isolates. The yellow areas mark distinct clonal groups (≤2 SNPs).

We compared the cgSNPs of isolates from the same sequence type across multiple wards. All cgSNP matrices are available in Supplementary Data S1. Among all sequence types, only ST3 and ST17 exhibited pairs with cgSNP differences ≤2 (Figure 5). Among ST3 strains, the pairwise cgSNP distances ranged from 1 to 201 (median 16). By integrating sampling time information, we identified 4 genetically highly related clone groups (cgSNP ≤2; sampling intervals of 15–115 days), consistent with the established time threshold for potential clonal transmission. The subsequent epidemiological investigation and hospitalization data analysis did not find confirmed evidence. Specifically, hosts of strain 160081, 160097, 160016, and 160017 had stayed in the Infectious Diseases Department at the time of the episode’s onset or before it; hosts of strain 160108 and 160130 stayed in the same ICU with admission times 115 days apart; strain 160057 and strain 160093, isolated from non-related patients in the Respiratory Department and Emergency Department, differed by only 1 cgSNP; strain 160031 and strain 160055 were sampled 52 days apart from different patients suffered hematological malignancy without spatial overlap. Notably, the wards of these patients were located far apart, in separate buildings. For ST17, the pairwise cgSNP differences ranged from 2 to 22 (median 13). Although one strain pair differed by 2 cgSNPs, the sampling interval was 129 days (>124 days), and their hosts had no shared ward exposure, which cannot indicate transmission. Overall, despite genetic and temporal proximity in some pairs, no definitive clonal dissemination due to close contact was confirmed within wards or across wards.

## Discussion

This study systematically delineated the clinical and pathogenic landscape of CDI in Southwest China in the post-COVID-19 era. Clinically, the disease burden remains substantial, driven by host susceptibility and healthcare-associated selection pressure. Genomic analysis revealed diverse sequence types and AMR profiles, confirming persistent regional-dominant lineages, alerting to potential clonal relatedness, and providing evidence for clinical treatment considerations. These findings align with the globally recognized spatiotemporal heterogeneity of *C. difficile* and uncover previously underdocumented region-specific transmission dynamics in the post-pandemic period. By integrating genomic and clinical data, this study enhances understanding of *C. difficile* epidemiological characteristics, resistance patterns, and transmission risks in healthcare settings, extending beyond previous studies that primarily relied on pre-2020 isolates and geographically limited cohorts.

Not surprisingly, our detection rate of CDI among suspected diarrhea inpatients was 5.4%. Over half of our patients were over 65 years old with typical high-risk medication exposures (such as PPIs and broad-spectrum antibiotics), consistent with two pre- and intra-pandemic studies from Chongqing (Dai et al., 2020; Cui et al., 2023) (Table 3) and previous global literature (Di Bella et al., 2024). In contrast to international studies reporting a higher proportion of females in CDI (Di Bella et al., 2024), males comprised the majority of CDI patients in this research, which aligns with the two previous studies from southwestern China (Dai et al., 2020; Cui et al., 2023) (Table 3). More importantly, the clinical burden of CDI in this region should not be underestimated. Lacking hypervirulent strains (no ST1 and only 1 ST11 were found), 20.4% of patients still developed severe infections, including two fulminant episodes. The causative strains from these patients were distributed across 8 distinct STs: ST3, ST2, ST54, ST37, ST81, ST42, ST17, and ST5 (Figure 2). The proportion of severe cases was comparable to the 21.5% reported in Zhejiang Province for the sampling years 2009–2016 (Xu et al., 2017) and lower than 41.4% in the United States (Drwiega et al., 2024), highlighting the combined regional differences in host susceptibility, strain genotype and toxicity, clinical management, and infection control strategies. Based on the above, there is a clear need to optimize antimicrobial and PPIs prescribing practices, refine risk stratification, and monitor elderly or immunocompromised patients more closely.

**Table 3:** Studies of *C. difficile* in Southwest China from 2018 to 2025.

Study	Location (population)	Sample collection period	Specimen number	Toxigenic isolates	Age ≥65 years (%)	Males (%)	Dominant toxigenic STs, % (*n*/total toxigenic isolates)	Toxin genotype, % (*n*/total toxigenic isolates)	FQ (*R*, %)	FDX (*R*, %)	MTZ (*R*, %)	VAN (*R*, %)
Liao et al. (2018)	4 sentinel hospitals in Yunnan province (46 million)	2013.01–2016.03	978	48	NR	NR	ST3, 31.3% (15/48); ST35, 20.8% (10/48); ST54, 18.8% (9/48)	*tcdA* + *tcdB* + *cdtA/B* − 97.9% (47/48); *tcdA* − *tcdB* + *cdtA/B* − 2.0% (1/48)	NR	NR	NR	NR
Dai et al. (2020)	A tertiary hospital in Chongqing, a provincial-level municipality (32 million)	2014.06–2016.03	122	38	60.5%	73.7%	ST2, 23.7% (9/38); ST37, 15.8% (6/38); ST54, 13.2% (5/38)	*tcdA* + *tcdB* + *cdtA/B* − 89.5% (34/38); *tcdA* − *tcdB* + *cdtA/B* − 7.9% (3/38); *tcdA* + *tcdB* + *cdtA/B +* 2.6% (1/38)	LVF 14.5%	NR	0%	0%
Gu et al. (2025)	4 sentinel hospitals in Yunnan province (46 million)	2013–2020	1,368	59	NR	NR	ST3, 25.4% (15/59); ST35, 20.3% (12/59); ST54, 15.3% (9/59)	*tcdA* + *tcdB* + *cdtA/B* − 100% (59/59)	CIP 13.0%	NR	0%	0%
Cui et al. (2023)	A tertiary hospital in Chongqing, a provincial-level municipality (32 million)	2021.01–2022.09	2,084	90	60.0%	56.7%	ST3, 27.8% (25/90); ST2, 11.1% (10/90); ST37, 10.0% (9/90)	*tcdA + tcdB + cdtA/B* − 76.7% (69/90); *tcdA* − *tcdB* + *cdtA/B* − 13.3% (12/90); *tcdA* + *tcdB* + *cdtA/B +* 10.0% (9/90)	MXF 62.2%	NR	0%	0%
This study	A tertiary hospital in Chengdu city (23 million) from Sichuan province (84 million)	2023.06–2024.05	2,917	109	51.0%	67.5%	ST3, 23.9% (26/109); ST81, 18.9% (20/109); ST2, 11.9% (13/109); ST54, 11.9% (13/109); ST37, 7.3% (8/109)	*tcdA* + *tcdB* + *cdtA/B* − 68.8% (75/109); *tcdA* − *tcdB* + *cdtA/B* − 25.7% (28/109); *tcdA* + *tcdB* + *cdtA/B +* 5.5% (6/109)	MXF 56.0%	0%	3.7%	0%

ST: Multilocus sequence type (MLST); FQ: fluoroquinolones; LVF: levofloxacin; FDX: fidaxomicin; MXF: moxifloxacin; CIP: ciprofloxacin; MTZ: metronidazole; VAN: vancomycin; R, resistance. Resistance percentages are shown as reported in the original studies. NR = not reported.

In this study, the predominant lineage was ST3, which consistently produces toxin A and B. This aligns with previous systematic reviews involving Chinese research before the pandemic (Wen et al., 2023), particularly with studies from other regions in Southwest China (Liao et al., 2018; Cui et al., 2023; Gu et al., 2025) (Table 3), further supporting that ST3 remains dominant in China in the post-pandemic era. Still, isolates that produce binary toxin and carry *cdtA/B* were rare, and no hypervirulent ST1/RT027 strains were identified. Instead, ST5, as a hypervirulent phenotype that appeared sporadically and was rarely reported in China (Shaw et al., 2020), was the main clone producing binary toxin prevalent in this region. ST11, occasionally identified in elderly patients in Beijing (Suo et al., 2017), was also isolated from one patient in this region. Since it was previously identified in animals in east China and farms were regarded as the reservoirs, we agree that both zoonotic and patient infections warrant ongoing surveillance (Zhang et al., 2019).

In this study, a notable shift in the dynamics of *tcdA* − *tcdB* + *cdtA/B*− strains was observed. ST37 has historically dominated in China and across Asia (Collins and Riley, 2018). But now, unlike other regions in Southwestern China (Dai et al., 2020; Cui et al., 2023) (Table 3), ST81 has replaced ST37 as the second-most prevalent clone in this region, following ST3. All our ST81 strains carried *gyrA* Thr82Ile and *gyrB* Ser366Ala/Asp426Val mutations (Figure 2), conferring universal resistance to moxifloxacin. A Japanese review (Senoh and Kato, 2022) and a study from Beijing (Cheng et al., 2020), both of which analyzed samples collected before the COVID-19 pandemic, reported an increased prevalence of ST81 with high fluoroquinolone resistance. Moreover, in the present study, approximately 20% of ST81 isolates also exhibited metronidazole resistance. Although they differ by only 1 allele, genomic and phenotypic comparisons suggested that ST81 had advantages over ST37 in sporulation, environmental adaptation, and AMR (Su et al., 2021). The persistent rapid expansion of ST81 with enhanced fitness and high AMR underscores the need for strengthened clinical surveillance to alert to a potential outbreak.

Despite strict prescription restrictions on fluoroquinolones at our hospital since 2017, resistance to moxifloxacin remained remarkably high at 56.0%, comparable to the intra-pandemic fluoroquinolone resistance levels of Chongqing (62.2%) (Cui et al., 2023), and significantly exceeding the pre-pandemic levels in southwestern China (13.0–14.5%) (Dai et al., 2020; Gu et al., 2025) (Table 3) and levels outside the Southwest China (16–43%) (Yang et al., 2020; Zhang et al., 2024). This aligns with the high exposure rate to fluoroquinolone antibiotics (second only to β-lactams) among CDI patients before the onset of illness, indicating that a history of fluoroquinolone usage remains a significant selective pressure. When integrated with molecular typing results (Figure 4), it becomes evident that both the dominant ST3 and clade 4, particularly ST81, contribute substantially to this trend, reinforcing the view that fluoroquinolone restriction stewardship should remain our critical intervention measure. Regarding metronidazole, the resistance rate in this study was 3.7%, higher than that reported in Shanghai (1.07%) but lower than some earlier data (15.7%) (Jin et al., 2017; Yang et al., 2020). In contrast, metronidazole resistance was not detected in other studies from southwestern China (Dai et al., 2020; Cui et al., 2023; Gu et al., 2025) (Table 3), collectively highlighting significant regional and temporal variations. No pCD-METRO plasmid was detected in this cohort, consistent with its reported global low prevalence (<0.2%) (Olaitan et al., 2023). Among the 14 ST81 isolates carrying P*nimB*^G^, only 4 demonstrated metronidazole resistance, consistent with a previous report of the genotype-phenotype discrepancy (Kolte and Nubel, 2024). Therefore, P*nimB*^G^ alone cannot determine the resistance phenotype, and its contribution may depend on other genetic and metabolic backgrounds. Notably, as shown in Figure 2, P*nimB*^G^ was observed exclusively among ST81 strains with the Thr82Ile substitution in *gyrA*, supporting the recent finding that strains carrying both mutations spread more rapidly (Olaitan et al., 2023). So, ST81 should be closely followed to prevent further dissemination, and genomic surveillance of *gyrA/gyrB* and *nimB* promoter variants is warranted.

Strains in our study harbored ARGs that confer resistance to multiple classes of antimicrobial agents. Of particular concern was the first detection of *cfr(B)* in one ST54 isolate in China. The *cfr* gene family confers cross-resistance to oxazolidinones (linezolid), phenicols, lincosamides, pleuromutilins, and streptogramin A, and has been demonstrated to be carried on mobile genetic elements with horizontal transfer potential. Previously, a Chinese study reported the presence of *cfr(B)* in ST37 and ST3 strains (Fu et al., 2025). Recent reports from animal sources have identified the *cfr(B)* gene in *C. difficile* (Brenciani et al., 2025). These findings indicate that the dissemination of *cfr(B)* is not restricted to a specific ST of *C. difficile*, and that *C. difficile* acts as a potential reservoir and vector for this resistance gene, underscoring its public health threat. It is therefore crucial to incorporate *cfr(B)* into ongoing integrated “One Health” surveillance efforts targeting human-animal-environment transmission pathways.

Integrating WGS with traditional epidemiological investigations facilitates the early identification of transmission. Incorporating genomic and temporal thresholds (e.g., ≤2 cgSNPs within 124 days) into the surveillance framework may provide an actionable early-warning tool for timely interventions. In this study, although genetically closely related strains were identified by cgSNP comparison and certain spatiotemporal overlaps were revealed by epidemiological investigations, no confirmed nosocomial transmission or distinct clonal dissemination due to close contact was found. However, the potential clonal dissemination should be on high alert through cryptic pathways from environmental reservoirs or community sources. Our findings support the importance of ongoing genomic surveillance and strengthening infection control, including enhanced cleaning and disinfection protocols, strict closed-loop management of patient transfers, and targeted screening of asymptomatic carriers or patients readmitted soon after discharge.

These post-pandemic dataset from Southwest China effectively addresses a gap in the existing literature, which has primarily focused on pre-2020 isolates or geographically restricted cohorts. Genomic findings integrated with detailed clinical data enable a more comprehensive interpretation of strain diversity, resistance profiles, and transmission risks in our hospital setting. However, several limitations should be acknowledged. First, this study only included CDI cases by the strict definition of combined toxin A/B assay and positive isolation of *C. difficile* strains, which offers high specificity but limited sensitivity, thereby underestimating the actual prevalence of *C. difficile* in this region. Second, the mechanisms underlying metronidazole resistance in ST81 in this study remain unclear and warrant further investigation. Third, the retrospective, single-center data could not fully capture the relationship between clinical outcomes and bacteriological features, necessitating validation in large, multicenter prospective cohorts. We call for establishing coordinated national-level multicenter surveillance networks to provide a more accurate assessment of the true burden of CDI in China and to harmonize surveillance strategies for the whole country.

## Conclusion

Our findings indicate that in the post-COVID-19 era in Southwest China, although no global hypervirulent strain is prevalent, the clinical burden of CDI cannot be overlooked, due to its disease severity. All *C. difficile* isolates are still susceptible to fidaxomicin and vancomycin. ST81 has significantly increased in prevalence as the second-most dominant strain type after ST3, generally exhibiting high-level fluoroquinolone resistance and partial metronidazole resistance, and warrants more attention. Core genome analysis could identify clone groups, prompting clinicians to assess whether cryptic clonal dissemination is present. Our sustained clinical case monitoring, genomic surveillance of isolates, and AMR pattern surveys integrate data on clinical cases and their pathogenic strains, providing further evidence to enhance infection control measures to prevent future clonal dissemination and supporting clinical management and public health decisions.

## Data Availability

The datasets presented in this study can be found in online repositories. The names of the repository/repositories and accession number(s) can be found in the article/Supplementary material.

## Footnotes

1
https://github.com/tseemann/snippy

2
https://github.com/tseemann/snp-dists
