# Supplementary material for: Clostridioides difficile infection cases and the causative strains in a large Chinese tertiary hospital in 2023–2024
Source: Front Microbiol. 2026 Feb 11;17:1749145. doi: 10.3389/fmicb.2026.1749145 (PMC12932564; doi:10.3389/fmicb.2026.1749145)
Supplement: Supplementary file 1 [file Table_1.DOCX]

**Supplementary Table 1** The GenBank accession numbers of 109 *C. difficile* strains.

| ID | Biosample_accession | Accession number |
| --- | --- | --- |
| 160002 | SAMN53259671 | SRR36065308 |
| 160004 | SAMN53259672 | SRR36065307 |
| 160007 | SAMN53259673 | SRR36065287 |
| 160010 | SAMN53259674 | SRR36065266 |
| 160011 | SAMN53259675 | SRR36065257 |
| 160012 | SAMN53259676 | SRR36065265 |
| 160013 | SAMN53259677 | SRR36065226 |
| 160014 | SAMN53259678 | SRR36065235 |
| 160016 | SAMN53259679 | SRR36065202 |
| 160017 | SAMN53259680 | SRR36065213 |
| 160018 | SAMN53259681 | SRR36065306 |
| 160020 | SAMN53259682 | SRR36065296 |
| 160021 | SAMN53259683 | SRR36065295 |
| 160022 | SAMN53259684 | SRR36065294 |
| 160023 | SAMN53259685 | SRR36065293 |
| 160024 | SAMN53259686 | SRR36065292 |
| 160025 | SAMN53259687 | SRR36065291 |
| 160026 | SAMN53259688 | SRR36065290 |
| 160027 | SAMN53259689 | SRR36065289 |
| 160030 | SAMN53259690 | SRR36065288 |
| 160031 | SAMN53259691 | SRR36065286 |
| 160032 | SAMN53259692 | SRR36065285 |
| 160033 | SAMN53259693 | SRR36065284 |
| 160034 | SAMN53259694 | SRR36065283 |
| 160035 | SAMN53259695 | SRR36065282 |
| 160036 | SAMN53259696 | SRR36065244 |
| 160037 | SAMN53259697 | SRR36065245 |
| 160038 | SAMN53259698 | SRR36065246 |
| 160039 | SAMN53259699 | SRR36065247 |
| 160040 | SAMN53259700 | SRR36065248 |
| 160041 | SAMN53259701 | SRR36065267 |
| 160042 | SAMN53259702 | SRR36065268 |
| 160043 | SAMN53259703 | SRR36065269 |
| 160044 | SAMN53259704 | SRR36065270 |
| 160046 | SAMN53259705 | SRR36065271 |
| 160048 | SAMN53259706 | SRR36065272 |
| 160049 | SAMN53259707 | SRR36065273 |
| 160050 | SAMN53259708 | SRR36065274 |
| 160051 | SAMN53259709 | SRR36065275 |
| 160052 | SAMN53259710 | SRR36065276 |
| 160053 | SAMN53259711 | SRR36065258 |
| 160054 | SAMN53259712 | SRR36065259 |
| 160055 | SAMN53259713 | SRR36065260 |
| 160056 | SAMN53259714 | SRR36065261 |
| 160057 | SAMN53259715 | SRR36065262 |
| 160058 | SAMN53259716 | SRR36065277 |
| 160059 | SAMN53259717 | SRR36065278 |
| 160060 | SAMN53259718 | SRR36065281 |
| 160061 | SAMN53259719 | SRR36065280 |
| 160062 | SAMN53259720 | SRR36065279 |
| 160063 | SAMN53259721 | SRR36065264 |
| 160064 | SAMN53259722 | SRR36065263 |
| 160065 | SAMN53259723 | SRR36065256 |
| 160066 | SAMN53259724 | SRR36065255 |
| 160067 | SAMN53259725 | SRR36065254 |
| 160068 | SAMN53259726 | SRR36065253 |
| 160069 | SAMN53259727 | SRR36065252 |
| 160070 | SAMN53259728 | SRR36065251 |
| 160071 | SAMN53259729 | SRR36065250 |
| 160074 | SAMN53259730 | SRR36065249 |
| 160075 | SAMN53259731 | SRR36065225 |
| 160076 | SAMN53259732 | SRR36065224 |
| 160077 | SAMN53259733 | SRR36065243 |
| 160078 | SAMN53259734 | SRR36065242 |
| 160079 | SAMN53259735 | SRR36065241 |
| 160081 | SAMN53259736 | SRR36065240 |
| 160082 | SAMN53259737 | SRR36065239 |
| 160083 | SAMN53259738 | SRR36065238 |
| 160084 | SAMN53259739 | SRR36065237 |
| 160085 | SAMN53259740 | SRR36065236 |
| 160086 | SAMN53259741 | SRR36065234 |
| 160088 | SAMN53259742 | SRR36065233 |
| 160089 | SAMN53259743 | SRR36065232 |
| 160091 | SAMN53259744 | SRR36065231 |
| 160092 | SAMN53259745 | SRR36065230 |
| 160093 | SAMN53259746 | SRR36065229 |
| 160094 | SAMN53259747 | SRR36065228 |
| 160095 | SAMN53259748 | SRR36065227 |
| 160096 | SAMN53259749 | SRR36065223 |
| 160097 | SAMN53259750 | SRR36065222 |
| 160098 | SAMN53259751 | SRR36065201 |
| 160100 | SAMN53259752 | SRR36065200 |
| 160102 | SAMN53259753 | SRR36065221 |
| 160104 | SAMN53259754 | SRR36065220 |
| 160105 | SAMN53259755 | SRR36065219 |
| 160107 | SAMN53259756 | SRR36065218 |
| 160108 | SAMN53259757 | SRR36065217 |
| 160110 | SAMN53259758 | SRR36065216 |
| 160111 | SAMN53259759 | SRR36065215 |
| 160113 | SAMN53259760 | SRR36065214 |
| 160114 | SAMN53259761 | SRR36065212 |
| 160115 | SAMN53259762 | SRR36065211 |
| 160116 | SAMN53259763 | SRR36065210 |
| 160117 | SAMN53259764 | SRR36065209 |
| 160118 | SAMN53259765 | SRR36065208 |
| 160120 | SAMN53259766 | SRR36065207 |
| 160121 | SAMN53259767 | SRR36065206 |
| 160122 | SAMN53259768 | SRR36065205 |
| 160123 | SAMN53259769 | SRR36065204 |
| 160124 | SAMN53259770 | SRR36065203 |
| 160125 | SAMN53259771 | SRR36065305 |
| 160126 | SAMN53259772 | SRR36065304 |
| 160127 | SAMN53259773 | SRR36065303 |
| 160128 | SAMN53259774 | SRR36065302 |
| 160129 | SAMN53259775 | SRR36065301 |
| 160130 | SAMN53259776 | SRR36065300 |
| 160131 | SAMN53259777 | SRR36065299 |
| 160132 | SAMN53259778 | SRR36065298 |
| 160133 | SAMN53259779 | SRR36065297 |
